# Understanding the Heterogeneous Impact of Innovation Efficiency on Urban Ecological Footprint in China

**DOI:** 10.3390/ijerph19106054

**Published:** 2022-05-16

**Authors:** Hui Zhang, Haiqian Ke

**Affiliations:** 1 Nanyang Institute of Technology, Fanli Business School, Nanyang 473000, China; zhanghuinylg@163.com; 2 Institute of Central China Development, Wuhan University, Wuhan 430072, China

**Keywords:** innovation efficiency, ecological footprint, dynamic threshold effect, night light data

## Abstract

Under the background of tightening resource constraints and a deteriorating ecological environment, innovation is aimed at saving energy, reducing consumption, abating pollution and achieving sustainable economic growth. This has gradually become an important way to improve industrial structure, competitiveness and environmental performance worldwide. In this study, we use the super-efficiency SBM model to calculate the innovation efficiency of 283 cities in China from 2009 to 2019. Then, based on the dynamic threshold regression model, we explore the impact of innovation efficiency on ecological footprint in innovative cities or non-innovative cities under different economic development levels. The main conclusions that can be drawn are as follows. (1) Within the research period, the influence of innovation efficiency on ecological footprint in China shows a negative double threshold feature, that is, increasing regional innovation efficiency has an inhibitory effect on ecological footprint. (2) For innovative cities, innovation efficiency has a strong inhibitory effect on ecological footprint, and it becomes stronger and stronger with the growth of night light data; but this inhibitory effect is gradually decreasing with improvement of economic development level in non-innovative cities. (3) Under the threshold of different levels of economic development, the number of scientific human resources, scientific financial resources, scientific information resources and scientific papers has a positive effect on ecological footprint, while the number of patent applications has a negative effect on ecological footprint.

## 1. Introduction

The concept of ecological footprint was first proposed by the Canadian ecological economist Rees in 1992 [1]. Ecological footprint specifically refers to space with biological productivity which could continuously provide resources or absorb waste, i.e., the geographical area needed to sustain one person or one region to live [2]. Ecological footprint realizes the unified description of various natural resources by introducing the concept of ecologically productive land, and further realizes the additivity and comparability of various types of ecologically productive land in various regions by introducing equilibrium factors [3]. More importantly, ecological footprint can objectively measure and compare the two-dimensional sustainability degree of time and space, so that people can clearly know how far is from a sustainability goal, which is helpful to monitor the implementation of a sustainability plan. Based on the advantages mentioned above, ecological footprint has a wide range of applications [4]. We can calculate the ecological footprint of individuals, families, cities, countries, realize vertical and horizontal comparative analyses of them, thereby promoting popularization of the concept.

In 2020, the range and frequency of human activities in different regions of the world decreased in different degrees due to the COVID-19 pandemic [5]. Nevertheless, in order to achieve long-term, stable, social and economic development, human beings are still continuously using various resources and putting certain pressures on the ecosystem. In fact, China’s ecological and environmental problems arise mainly from the process of advancing industrialization. China’s increasingly excessive consumption of resources, is giving rise to a bottleneck of resource depletion and ecological environment deterioration. However, innovation aims at saving energy, reducing consumption, reducing pollution and achieving sustained economic growth. The more advanced the technology, the more environmentally friendly it tends to be. Therefore, innovation has gradually become an important path for countries around the world to improve industrial structure, competitiveness and environmental performance [6]. As China enters this period of transformation and development, China’s economy is gradually shifting from relying on traditional factors towards innovation. That is, promoting innovation levels in the future is an important driving force for China’s sustainable development.

Innovation is actually a complex nonlinear process with multi-factor inputs and outputs. The input of innovative resources does not necessarily lead to an equivalent innovative output. Innovation efficiency, as an important indicator to measure the level of regional innovation, refers to the efficiency of distribution and use of various scientific and technological resources in different fields of scientific and technological activities, and it is also the result of cooperation and interaction among all components of the innovation system [7,8,9]. Additionally, innovative cities are different from non-innovative cities in the following two aspects. Firstly, innovative cities are the pilot objects of national innovation-driven development strategy, because promoting the innovation level of cities is not only the result of market selection, but also the result of national participation and government strategic guidance. Secondly, by increasing the government’s input of innovation resources, innovative cities can improve the gathering ability of urban innovation elements and guarantee the supply of knowledge elements in innovation activities, which is conducive to promoting the formation of urban innovation systems with enterprises as the main bodies and improvement in the level of urban innovation [10,11]. Then, will improvement in innovation efficiency help to promote sustainable development of regional society, economy and ecology? How will regional innovation efficiency affect the ecological footprint between innovative cities and non-innovative cities? To address these questions, this paper uses the super-efficiency SBM model to measure the innovation efficiency of Chinese cities from 2009 to 2019, uses DMSP/OLS nighttime light data to characterize regional socioeconomic development level, and analyzes the impact of China’s innovation efficiency on ecological footprint based on the dynamic threshold regression model. This re-examines the relationship between innovation efficiency and ecological footprint, broadens the research ideas in this field, and, thereby, exploring the deep integration path of regional innovation development and green development in China.

## 2. Literature Review

Ecological footprint refers to the traces left by human impact on the natural ecological environment. Therefore, the larger the ecological footprint, the more serious the damage to the ecological environment [12]. Since 1970, the total global ecological footprint has begun to exceed the earth’s carrying capacity, and it has been on the rise year by year. China’s ecological footprint has shown a rapid upward trend since 2000. Data provided by Global Footprint Network (GFN) in 2020 show that although China ranks 66th in the world in terms of ecological footprint per capita, the rate of resource consumption in China has seriously exceeded the rate of resource renewal and China’s rate ranks first in the world with a total ecological footprint of 5.35 billion global hectares [13]. It shows enormous pressure on China’s ecological environment. Therefore, it is necessary to conduct continuous tracking studies on China’s ecological and environmental problems.

In fact, China’s ecological footprint basically shows a “stepped” spatial distribution pattern with the highest in the east, the second highest in the center, and the lower in the west. Jia et al. (2009) used panel data and the extended STIRPAT (Stochastic Impacts by Regression on Population, Affluence, and Technology) model to decompose the variability of ecological footprints across eight regions of China, showing that the influences of population size, economic growth, energy efficiency, and industrial structure on ecological footprint were significant [14]. Chen & Chen (2016) analyzed the changes of urban ecological footprint in China, and their influencing factors as well, and the results showed that population growth, income level, energy efficiency, industrial structure, etc. were important factors causing differences in provincial ecological footprints, while with higher economic levels, the ecological footprints of the eastern regions were generally higher than those of the central and western regions [15]. Therefore, innovation may have different degrees of impact on the quality of the ecological environment, due to the differences in levels of regional economic development. Notably, Kabir et al. (2021) found that the inhibitory effect of technological innovation on pollutant emission reduction in China was enhanced by the level of regional economic development for the following reasons [16]. First, higher economic level could provide effective financial support, thereby increasing the technological innovation level, which is conducive to the vigorous development, promotion and utilization of clean energy, effectively reducing carbon emissions, curbing pollutant emissions and alleviating pressure on the ecological environment. Second, with improvement of the level of economic development, social awareness of environmental protection is relatively enhanced, and people’s consumption preferences gradually shift from focusing on the price of the final product to attaching importance to aspects such as environmental protection and energy conservation, which has a positive impact on the ecological environment [17]. It is essentially the same as the content of the environmental kuznets “inverted U-shaped” curve (EKC), proposed by Grossman & Krueger (1995), and the porter hypothesis (Porter, 1990) [18,19]. That is, with improvement of the level of economic development, innovation plays a more and more important role in improving the quality of the ecological environment. So, does innovation efficiency follow the same rule for ecological footprint? It needs to be further explored.

In 2008, the National Development and Reform Commission of China approved Shenzhen as the first national pilot innovative city, and the number of innovative cities has been increasing since then. So far, 78 innovative pilot cities have been approved by the Ministry of Science and Technology [20]. The pilot innovative city aims to carry out the construction practice of innovative cities with local characteristics, according to the endowment conditions of the city’s own resource base, development level, location advantages, industrial characteristics and so on. The Chinese government has clearly proposed to build innovative cities into cities with strong independent innovation ability, outstanding scientific and technological support, high levels of sustainable economic and social development, and remarkable regional radiation. Therefore, the inclination of innovation policy enables innovative cities to obtain more abundant innovation factor resources [21]. With the superposition of policy effects, innovative cities have effectively enhanced the input–output efficiency of innovation. At the same time, in the process of implementation of innovation policies, they will continue to correct and improve themselves, so that the implementation of innovation policies will be more targeted and compatible, and more able to meet the realistic needs of independent innovation in innovative cities, and will have stronger roles in promoting the efficiency of their own innovations. However, non-innovative cities will find it difficult to improve their efficiencies, in terms of innovation input and output, due to lack of relevant policy support and lack of effective construction of innovation platforms. Existing research still lacks attention regarding this issue. That is, there may be a difference in the impact of innovation efficiency on ecological footprint between innovative cities and non-innovative cities.

During the process of continuous expansion of urban areas, Malthus (1798) was the first to point out that the main reason for lack of regional resources comes from limits imposed by growth of population [22], which is also the starting point for people to pay attention to the impact of population scale on the environment. Commoner (1971) believes that the development of industrial technology is the primary cause of environmental quality deterioration [23]. At the same time, the “population growth theory” proposed by Ehrlich & Holdren (1971) believes that “compared with sophisticated management technology, the oversize population is the deep-rooted cause of ecological environment pressure” and emphasizes that population growth is the most important cause of environmental deterioration [24]. Also, innovation could influence urban sustainability in different ways [25,26]. Therefore, the marginal contributions of this paper are as follows: (1) As the city is the extension and carrying space of national innovation and regional innovation, based on 283 cities from 2009 to 2019, this paper provides a systematic study on the impact of ecological footprint from a new perspective of innovation efficiency, instead of innovation itself. (2) Taking nighttime light data as the threshold variable is more comprehensive and objective, as it can measure real economic growth, and also tests economic agglomeration, urbanization, population mobility, and energy consumption. (3) This paper verifies whether the government’s policy support for innovation will have an impact on the relationship between innovation efficiency and ecological footprint, and what impact it will have. The conclusions will help relevant departments to formulate specific and differentiated policies, so as to promote China’s sustainable development.

The rest of the paper is organized as follows: the third part presents the model and data sources, the fourth part provides the analysis of regression results, and, finally, we present the conclusions and discuss the policy implications.

## 3. Data and Model

### 3.1. Variable Description

① Explained variable (ecological footprint): in the calculation of ecological footprint, various resources and energy consumption items are converted into six types of biological production area, including cultivated land, grassland, woodland, construction land, fossil energy land and ocean (water area). Cultivated land is the most productive land type, providing most of the biomass used by human beings. The values of equilibrium factors, given by various institutions and researchers in different years, are relatively stable, with little differences. Therefore, this paper selects the equilibrium factor data provided by “Global Footprint Network” (GFN) in 2018: cultivated land 2.52, grassland 0.43, woodland 1.28, water area 0.35, energy land 1.28, and construction land 2.52 [27]. The formula is as follows:
  (1) EF=N×ef
  (2) $$\mathrm{ef}=\sum_{j=1}^{6}\sum_{i=1}^{n}(r_{j}a_{i})=\sum_{j=1}^{6}\sum_{i=1}^{n}(r_{j}\times c_{i}/p_{i})\;j=(1,2,3\ldots 6)$$

In Equation (1), *EF* is the total ecological footprint of the region, *ef* is the ecological footprint per capita of the region, and *N* is the population in the region. In Equation (2), *i* is the category of consumption resource, *a_i_* is the ecologically productive land occupied per capita converted from the world average product of the *i*th consumption resource, *c_i_* is the product per capita of the *i*th consumption resource, *p_i_* is the world average product of the ecologically productive land producing the *i*th consumption resource, and *r_j_* is the equilibrium factor of the *j*th ecologically productive land. There are six ecologically productive lands.

② Core explanatory variable (innovation efficiency): this paper mainly measures innovation efficiency from the perspective of input and output of scientific and technological resources. The inputs in scientific resources are mainly reflected in the allocation of scientific human resources, financial resources, scientific and technological information resources, and other elements. Among these, scientific human resources are represented by the full-time equivalent of research and development personnel, an indicator that reflects the ability of regional talent attraction. The scientific financial resources are represented by the internal expenditure of Research and Development funds, an indicator that reflects the level of regional support for scientific and technological activities. The development level of regional scientific information resources is reflected by the number of internet users. In terms of the outputs of scientific resources, the number of scientific papers and patent applications represent scientific achievements. Considering that the number of patent grants is highly uncertain, due to the influence of human factors, such as patent granting agencies, the number of patent applications accepted can better reflect the true level of scientific resource output than the number of patent grants [28,29,30,31].
③ Threshold variable (night light data): the current research on the temporal and spatial pattern of economic development mainly relies on statistical data. However, statistical data have the shortcomings of inconsistent caliber and low spatial resolution, which make it difficult to accurately portray the pattern characteristics of regional economic development. Night light data detects bright light emitted from the Earth’s surface and is an effective data source for studying human activities. DMSP/OLS data are currently one of the most widely used night light data, and have been used in studies for population estimation, electricity consumption estimation, urban sprawl monitoring, etc. In recent years, economists have introduced night light data into the economic statistical framework to measure the activity and distribution characteristics of economic activities, because it has the advantages of easy access, wide coverage and high correlation with human social and economic activities [32,33]. Therefore, this paper uses the stable light data from 2009 to 2019 as the indicator of regional economic development level, and then mainly explores the impact of innovation efficiency on ecological footprint under different economic levels.
④ Control variable: this paper selects the following six control variables from the perspective of economy, society and environment (shown in Table 1): total foreign direct investment (units of 10,000 RMB), proportion of tertiary industry (units of %), consumption of urban residents (units of 10,000 RMB), consumption of rural residents (units of 10,000 RMB), number of college teachers (units of thousand people) and pollution control investment / GDP (units of %) [34,35,36,37,38].

### 3.2. Data Sources

Considering the availability and representatives of data, this paper takes 283 cities in China as the research object (15 western cities out of 298 prefecture level cities are excluded, due to lack of necessary statistical data). The basic data for measuring ecological footprints were obtained from relevant yearbooks, such as China Urban Statistical Yearbook, China Statistical Yearbook, China Environmental Statistical Yearbook, China Forestry Statistical Yearbook, China Social Statistical Yearbook, Compilation of Foreign Resource, Energy and Environmental Statistics, China Rural Statistical Yearbook, and Compilation of Statistical Information of New China’s Six Decades from 2010–2020. The basic data for measuring innovation efficiency are obtained from the 2010–2020 China Science and Technology Statistical Yearbook and the official website of the National Bureau of Statistics of the People’s Republic of China, etc. In this paper, the data of DMSP/OLS from 2009 to 2019 are used as the data of urban night lights. The basic data of relevant control variables are obtained from relevant yearbooks 2010–2020, such as China Urban Statistical Yearbook, China Statistical Yearbook, China Environmental Statistical Yearbook and China Rural Statistical Yearbook [39].

### 3.3. Model

It is hard to satisfy the strict assumptions in the process of practical application of Hansen’s (1999) static panel threshold model, and there may be multicollinearity, significance bias and endogeneity among variables in the model [40]. In order to address some of the shortcomings of the static threshold model, Kremer et al. (2009; 2013) subsequently proposed a dynamic threshold model that incorporates the lagged terms of the explained variables, and this dynamic model puts lagged terms of explained variables into the dynamic threshold model as explanatory variables, thereby solving the endogeneity and lagging problems of the variables to the greatest extent [41,42]. Furthermore, based on the SIRPAT model (equation), we propose the following Equation (3) [24,43], which first assumes that there is only one threshold, but that there is also the possibility of two and more thresholds as well. In order to analyze more accurately, we set the double threshold model and the triple threshold model, as shown in Equations (4) and (5). Similarly, the formula for the double threshold test and the triple threshold test are as follows, and the specific models are as follows.

(3) $$I_{\mathrm{it}}=aP_{\mathrm{it}}^{b}A_{\mathrm{it}}^{c}T_{\mathrm{it}}^{d}e$$

(4) $$\begin{array}{l} {\mathrm{lnEF}}_{\mathrm{it}}=\alpha X_{\mathrm{it}}+\beta_{1}{\mathrm{lnie}}_{\mathrm{it}}\times I(T_{\mathrm{it}}\leq \delta_{1})+\beta_{2}{\mathrm{lnie}}_{\mathrm{it}}\times I(\delta_{1}<T_{\mathrm{it}}\leq \delta_{2})\\ +\beta_{3}{\mathrm{lnie}}_{\mathrm{it}}\times I(T_{\mathrm{it}}>\delta_{2})+\beta_{4}{\mathrm{lnEF}}_{\mathrm{it}-1}+\beta_{5}{\mathrm{lnEF}}_{\mathrm{it}-2}+C+\varepsilon_{it} \end{array}$$

(5) $$\begin{array}{l} {\mathrm{lnEF}}_{\mathrm{it}}=\alpha X_{\mathrm{it}}+\beta_{1}{\mathrm{lnie}}_{\mathrm{it}}\times I(T_{\mathrm{it}}\leq \delta_{1})+\beta_{2}{\mathrm{lnie}}_{\mathrm{it}}\times I(\delta_{1}<T_{\mathrm{it}}\leq \delta_{2})\\ +\beta_{3}{\mathrm{lnie}}_{\mathrm{it}}\times I(\delta_{2}<T_{\mathrm{it}}\leq \delta_{3})+\beta_{4}{\mathrm{lnie}}_{\mathrm{it}}\times I(T_{\mathrm{it}}>\delta_{3})+\beta_{5}{\mathrm{lnEF}}_{\mathrm{it}-1}+\beta_{6}{\mathrm{lnEF}}_{\mathrm{it}-2}+C+\varepsilon_{it} \end{array}$$

In the above equation, *I* is the environmental impact, *P* is the population, *A* is the affluence level, *T* is the technology level; *lnEF_it_* is the ecological footprint of the *i*th region in year *t*, *lnEF_it_*_−1_ is one period lagged of the ecological footprint of the *i*th region in year *t*, *lnEF_it_*_−2_ is two periods lagged of the ecological footprint of the *i*th region in year *t*, *lnie_it_* is the core explanatory variable, *T* is the threshold variable(night light data), *δ* is the fixed threshold value, *α* is the influence coefficient of *lnie_it_* on the explained variable, *β*_1_ and *β*_2_ are the influence coefficients of the core explanatory variable *lnie_it_* on the explained variable when *T_it_* ≤ *δ*, *T_it_* > *δ* respectively, *C* is a constant term, *ε_it_*~(0, *σ*) is a random disturbance term, *I* is an indicative function. The value of *I* depends on whether the condition in parentheses holds, and it takes the value of 1 when the corresponding condition holds, otherwise it takes the value of 0.

## 4. Empirical Results

### 4.1. Analysis of Threshold Test Results for 283 Cities in China

The Hausman significance test of the model showed that the original hypothesis was rejected, so the fixed effects model was chosen for analysis. Based on the fixed effects model, ecological footprint is used as the explained variable; nighttime light data (reflecting the level of economic development) is used as the threshold variable to measure the impact of innovation efficiency on ecological footprint under different levels of economic development. The core explanatory variables are tested in turn. The sampling method is the bootstrap method with 300 times. The test results are shown in Table 2.

From Table 2, it can be seen that innovation efficiency, scientific human resources, and information resources pass the single threshold test at the significance level of 1%, the number of scientific papers passes the single threshold at the significance level of 5%, and the number of patent applications does not pass the single threshold test. Innovation efficiency, scientific financial resources, scientific human resources, scientific information resources, the number of scientific papers and patent applications pass the double threshold test at the significance level of 1%, respectively. Moreover, scientific financial resources, scientific human resources, scientific information resources, the number of scientific papers, and the number of patent applications are all collinear with other thresholds, which do not meet the requirements. In addition, only scientific human resources passed the three-threshold test at the 1% significance level. Therefore, this study adopts the double-threshold test for the core variables.

From Table 3 and Table 4, it can be seen that under the dynamic threshold model with one period lagged and two periods lagged of ecological footprint as the explained variables, there is a certain endogenous association between one period lagged and two periods lagged of the ecological footprint. The coefficients of *lnEF_it_*_−1_ and *lnEF_it_*_−2_ are significantly positive, indicating that the endogeneity caused by the omitted variables is controlled, to some extent, by using the dynamic threshold model. In model (1), when the level of economic development is below the first threshold of 4.952 (the natural logarithm of the value of urban nighttime lights), the elasticity coefficient of innovation efficiency to ecological footprint is −0.1204; when the level of economic development is between the first threshold of 4.952 and the second threshold of 6.966, the elasticity coefficient of innovation efficiency to ecological footprint becomes −0.0953; when the level of economic development exceeds the second threshold of 6.966, the elasticity coefficient of innovation efficiency to ecological footprint is −0.0703, and all of these show the significance at the 1% level. Therefore, the impact of increased innovation efficiency on ecological footprint is characterized by a negative double threshold. This is mainly due to two reasons. First, when innovation is more efficient, the level of technological innovation is further enhanced. While promoting economic growth, it can improve production efficiency, improve energy utilization efficiency, and promote the development of new energy sources, thereby reducing the occupation of natural resources and alleviating pressure on the ecological environment brought about by rapid economic development. Second, new environmental protection technologies promote the birth of more environmentally friendly products, and reduce the level of pollutant emissions from enterprises, hence reducing the degree of industrial pollution. Therefore, the improvement of innovation efficiency has a significant inhibitory effect on ecological footprint. However, it should be noted that with the improvement of threshold variables (economic development level), the inhibitory effect of innovation efficiency on ecological footprint is gradually weakened, because the absolute value of its coefficient becomes smaller and smaller. The main reason for this phenomenon is that China’s economy is at a critical stage of transition from factor-driven to innovation-driven, and economic growth still relies mainly on the exploitation and use of natural resources, which undoubtedly puts relatively high pressure on the ecological environment, which is also confirmed by the sustained growth rate of the ecological footprint of Chinese cities.

From the perspective of innovation inputs, in model (2), the impact of scientific human resources on ecological footprint under different economic development levels generally firstly shows an inhibition effect and then a promotion effect. When the economic development level is below the first threshold of 4.662, the elasticity coefficient of scientific human resources to ecological footprint is 0.0405; after economic development level crosses the second threshold of 5.854, the elasticity coefficient of scientific human resources to ecological footprint is 0.0470. It is due to the fact that, as the level of economic development increases, more and more people provide sufficient labor and creativity for the socio-economic development of cities, but this also means more resource consumption and energy consumption, which put more pressure on the ecological environment. Since the process of consumption is the process of generating an ecological footprint, theoretically, people’s consumption of food, clothing, supplies, transportation and other consumption will undoubtedly exert pressure on limited resources and the environment, and thus increase ecological footprint. In model (3), the degree and direction of the impact of scientific financial resources on ecological footprint are both positive under different economic development level thresholds. This indicates that, as investment of scientific financial resources increases, the intensity of infrastructure facilities, related to the improvement or construction of the innovation system, increases, which means that more and more cultivated land is occupied as construction land, and this process of occupation will produce more pollution, thus increasing ecological footprint. It also shows that increase in ecological footprint cannot be effectively curbed by only relying on increase in investment of scientific financial resources, without focusing on the efficiency of the innovation system. Similarly, in model (4), as the level of economic development increases, the increase of scientific information resources has a certain promotion effect on the level of ecological footprint. The possible reason is that scientific information resources need to match other information resource sharing platforms as carrier support. If there is a lack of collaboration and effective communication between platforms, scientific information cannot effectively enhance the level of innovation; therefore, it is difficult to alleviate pressure on the ecological environment, showing the promotion effect on ecological footprint.

In terms of the output of innovation, in model (5), the number of scientific papers has a significant promoting effect on ecological footprint. This indicates that knowledge innovation contributes to the level of ecological footprint of a region. However, with the improvement of the level of economic development, the coefficient moves from 0.4441 to 0.0294, indicating that this promotion effect is gradually weakened. The reason is that there is not only a certain lag in the transformation of knowledge innovation into technological innovation, but also the transformation process depends on the diffusion path and intensity of knowledge innovation. The higher the intensity of knowledge diffusion between regions, the stronger the knowledge heterogeneity, and the stronger the improvement effect on innovation efficiency, so that the growth of ecological footprint can be better restrained. In model (6), the number of patent applications has a significant inhibitory effect on ecological footprint. When the level of economic development is below the first threshold of 4.676, the elasticity coefficient of patent applications to ecological footprint is −0.0443. When the level of economic development is between the first threshold of 4.676 and the second threshold of 7.757, the elasticity coefficient of patent applications to ecological footprint becomes −0.0507. When economic development level exceeds the second threshold of 7.757, the elasticity coefficient of patent applications to ecological footprint changes to −0.9126. This is because the increasing number of patent applications helps to improve innovation capacity, which leads to cost reduction and product quality optimization, while reducing energy consumption, saving resources and reducing waste generated, thus alleviating ecological pressure. Therefore, patent applications exhibit a significant inhibitory effect on ecological footprint.

Regarding the control variables, increase in scale of foreign investment can significantly promote more advanced environmental protection technologies, thus effectively curbing ecological footprint. The service industry, represented by tertiary industry, has a significant inhibitory effect on ecological footprint compared with consumption and the amount of waste generated by the primary and secondary industries, which indicates that the Chinese government needs to urgently and vigorously develop tertiary industry. The consumption level of urban residents and rural residents generally show a significant role in promoting ecological footprint, which is also in line with the speculation at the beginning of the article. As the consumption level increases, ecological pollution will increase accordingly. However, the consumption level of urban residents in models (1), (5), and (6) shows an inhibitory effect. This may be because the improvement in innovation is more likely to occur in urban areas, and urban residents often have stronger environmental awareness. Thus, despite increase in consumption levels of urban residents, ecological problems can still be mitigated to some extent. Although the influence coefficient of education level on ecological footprint is negative, it is not significant. The higher the proportion of pollution control investment in GDP, the greater the investment in ecological environment governance in a region, which effectively controls the degree of pollution generated. Therefore, it shows a significant inhibitory effect on the ecological footprint in all models.

### 4.2. Analysis of the Threshold Test Results for Innovative Cities

The term ‘innovative city’ refers to a city driven by innovative elements, such as science and technology, knowledge, manpower, culture and system, having high-end radiation and playing a leading role for other regions. In 2008, China’s National Development and Reform Commission approved Shenzhen as the first national innovative city pilot. Since then, the number of innovative cities has been increasing, and 78 of them have been approved by the Ministry of Science and Technology as pilot cities. Therefore, this paper takes innovative cities and non-innovative cities as research objects to carry out the regression analysis of the dynamic threshold model; including 75 innovative cities and 208 non-innovative cities. Due to the limited availability of data, Lhasa, Shihezi and Changji are not within the scope of this study, so there are 75 innovative cities and 205 non-innovative cities.

It can be seen from the results in Table 5 and Table 6 that one period lagged and two periods lagged of the ecological footprint of innovative cities are explained variables. There is a certain endogenous correlation, that is, the coefficients of *lnEF_it_*_−1_ and *lnEF_it_*_−2_ are significantly positive. This shows that the dynamic threshold model is used to control the endogeneity caused by missing variables to a certain extent. In model (1), when the level of economic development is below the first threshold of 3.025, the elasticity coefficient of innovation efficiency to ecological footprint is −0.1749. When the level of economic development is between the first threshold of 3.025 and the second threshold of 4.767, the elasticity coefficient of innovation efficiency to ecological footprint becomes −0.2449. When the level of economic development exceeds the second threshold 4.767, the elasticity coefficient of innovation efficiency to ecological footprint is −0.3066, and all of these show the significance at the 1% level, that is, the inhibitory effect of innovation efficiency in innovative cities on ecological footprint gradually increases with economic growth. This indicates that the improvement of innovation efficiency of innovative cities can better promote the level of technological innovation in the region, while improving resource utilization, increasing the development of new energy technologies and reducing the emission of pollutants in the production process, thus producing an increasingly strong inhibitory effect on ecological footprint. The analysis results of the other five control variables are basically consistent with the regression results in Table 4, except that the coefficient and significance of *lnfdi* are significantly improved. This may be because with the improvement of innovation efficiency in innovative cities, the urban population centered on R and D personnel, and supported by various service workers, accelerates to gather in innovative cities. When more people tend to flow from non-innovative cities to innovative cities, the accumulation of human capital brings an increasing level of technological innovation. Since more advanced technology tends to be greener, it has a certain inhibitory effect on ecological footprint.

### 4.3. Analysis of the Threshold Test Results for Non-Innovative Cities

The results in Table 7 and Table 8 show that there is a certain endogenous correlation between one period lag and two periods lag of ecological footprint in non-innovative cities in the dynamic threshold model with the lag phase I or lag phase II of the ecological footprint as explained variables. That is, the coefficients of *lnEF_it_*_−1_ and *lnEF_it_*_−2_ are significantly positive. It shows that the endogeneity caused by omitted variables is controlled to some extent in this paper by using a dynamic threshold model. In model (1), when the level of economic development is from the first threshold of 6.038 to the second threshold 6.945, the coefficients of innovation efficiency to ecological footprint changes from –0.3002, –0.1596 to –0.0980, and all of them show significance at the 1% level, that is, the inhibitory effect of innovation efficiency on ecological footprint of non-innovative cities gradually decreases with growth of urban night light data. This shows that as the economic level of non-innovative cities increases, the inhibitory effect of innovation efficiency on ecological footprint becomes weaker and weaker, which is similar to the regression results obtained by 283 cities across the country. It is worth noting that the inhibitory effect of increase in the number of patent applications on ecological footprint diminishes with increase in the nighttime light data. The possible reasons for this are that the innovation transformation platforms in non-innovative cities are not perfect, the infrastructure of the innovation system is relatively lacking, which make it difficult to promote the actual use of patents, and therefore it is difficult to significantly improve the level of technological innovation, which, in turn, leads to a weak inhibitory effect on ecological footprint. Therefore, in future development of non-innovative cities, we should concentrate limited R and D resources on advantageous inputs and outputs, make key breakthroughs in bottleneck problems, and make efforts in management of the innovation system, so as to promote overall innovation efficiency of non-innovative cities. It is worth noting that increase in number of scientific papers has a significant inhibitory effect on ecological footprint in innovative cities, while promoting the growth of ecological footprint in non-innovative cities. This distinct result shows that the impact of scientific papers on ecological footprint largely depends on the transformation efficiency of urban innovation platforms and the degree of optimization of the innovation environment. That is, the higher the transformation efficiency of the innovation platform and the better the innovation environment, the more beneficial it is for scientific papers to exert their inhibitory effect on ecological footprint. The analysis results of the other control variables are basically consistent with the model results in Table 4.

## 5. Conclusions

This paper uses the SBM model to calculate the innovation efficiency of 283 cities in China from 2009 to 2019. Taking night light data as the threshold variable, this paper first systematically investigates the impact of innovation efficiency on ecological footprint in China. Then it further analyzes whether innovation support policy will change this impact. The following conclusions are as follows:

(1) The impact of China’s innovation efficiency on ecological footprint presents a negative double-threshold feature. The improvement of innovation efficiency can effectively restrain the increase of ecological footprint, but, with improvement of economic development level, this restraining effect is gradually weakened. Similarly, non-innovative cities follow this pattern as well. This shows that, although innovation efficiency has slowed down the increasing speed of ecological footprint, to a certain extent, it still has not changed the fact that China’s ecological footprint continues to grow. Therefore, China needs to formulate different strategies for cities to promote innovation efficiency under different economic development levels, actively open up the innovation chain between cities, strengthen close cooperation between industries, universities and research institutes among cities, and comprehensively promote improvement of innovation efficiency and innovation level of Chinese cities. In addition, it is necessary to change China’s economic growth mode and industrial structure, so as to gradually reduce dependence on natural resources; strengthen development of new energy and open up new energy supply channels, change traditional consumption patterns and vigorously advocate “green consumption”, thereby effectively reducing ecological footprint.
(2) Compared with non-innovative cities, the improvement of innovation efficiency of 75 innovative cities in China has a stronger inhibitory effect on ecological footprint, and this inhibitory effect becomes stronger and stronger with increase of night light data. Therefore, it is necessary to improve the level of regional openness of non-innovative cities, improve the ability of information exchange between regions, reduce administrative barriers in regional innovation systems, strengthen cooperation in scientific innovation, promote linkage of scientific facilities, interoperability of innovation platforms and circulation of talent resources, provide policy encouragement and support, build a more complete innovation system, and create a higher-quality innovation highland. It is also necessary to integrate various innovative elements of innovative cities, strengthen exchanges and cooperation between scientific resources, enterprises and governments, within and between regions, achieve good synergies, continuously optimize regional innovation environments, and stimulate innovation vitality, accelerate the transfer and transformation of scientific and technological achievements, and then strengthen the inhibitory effect of innovation efficiency improvement on ecological footprint.
(3) Under different thresholds of economic development levels, scientific human resources, scientific financial resources, scientific information resources, and the number of scientific papers all show a promoting effect on ecological footprint. Therefore, Chinese cities should improve the overall level of scientific human resources, in terms of quantity and quality; optimize the investment structure of scientific financial resources to form a multi-channel and multi-level effective constraint systems; promote the construction of a more efficient science and technology information sharing platforms; and explore feasible paths for efficient diffusion and transformation of knowledge innovation into technological innovation. It should be noted that while improving China’s overall innovation efficiency, it is necessary to use as little resources as possible in the construction of the innovation system, and reduce the growth effect on ecological footprint.
(4) The number of patent applications has a negative effect on ecological footprint. Therefore, we need to speed up the efficiency of approval from patent application to licensing, so that patents can be used more efficiently as technology in the production process. From the perspective of environmental protection, patents can be roughly divided into green and non-green categories. The emphasis and promotion of “green innovation and green patent” and the introduction of environmental performance indicators will further strengthen the suppression effect of patents on ecological footprint.

The conclusion of this paper is consistent with some scholars, that is, the improvement of innovation level or innovation efficiency has a significant inhibitory effect on ecological footprint [38,44,45,46]. However, some scholars believe that the improvement of innovation level has no significant effect on ecological footprint [47,48]. Furthermore, there are many factors affecting the improvement of innovation efficiency, which can be divided into two categories: internal efficiency, that is, the efficiency of the internal management process of each subsystem, and external efficiency, that is, the efficiency of the transaction process, such as cooperation and communication between subsystems. Innovation efficiency first depends on internal efficiency of the innovation system, and also depends on the efficiency of cooperation and communication between subsystems. The combination of the two ensures innovation becomes an organic whole. This paper lacks further differentiation of different forms of innovation efficiency. Future studies can further explore whether there is heterogeneity in the impact of innovation efficiency on ecological footprint at different stages.

## Figures and Tables

**Table 1:** Selection and description of variables.

Variable Type	Variable Group	Symbol	Description
explained variables	ecological footprint	*lnEF*	biologically productive land area necessary to sustain human resource consumption and waste absorption
core explanatory variables	innovationefficiency	*lnie*	allocation and utilization efficiency of various scientific and technological resources in different subjects, fields, processes, space and time of scientific and technological activities
(input of scientific resources)	scientific human resources	*lnhr*	full-time equivalent of R and D personnel
	scientific financial resources	*lnfr*	internal expenditure of R and D funds
	scientific information resources	*lnir*	number of international Internet users
(output of scientific resources)	number of sci-tech papers	*lnpaper*	number of science-technology papers published
	number of patent applications	*lnpatent*	number of patent applications accepted
threshold variables	nighttime light data	*lnnl*	2009–2019 DMSP/OLS data
control variables	foreign direct investment	*lnfdi*	total amount of foreign direct investment in a certain period of time
	proportion of the tertiary industry	*lnthird*	ratio of service industry to GDP
	consumption of urban residents	*lnurbanc*	the total consumption expenditure of urban residents on food, clothing, household equipment, supplies and services, health care, transportation and communication, education, entertainment and services, housing, and miscellaneous goods and services
	consumption of rural residents	*lnruralc*	total consumption expenditure of rural residents on food, clothing, household equipment, supplies and services, health care, transportation and communication, education, entertainment and services, housing, and miscellaneous goods and services
	Number of college teachers	*lnedu*	number of teachers in urban institutions of higher learning
	pollution control investment/GDP	*lnpollu*	the ratio of pollution control investment to GDP

**Table 2:** The threshold effect test.

Innovation Indicators	Innovation Efficiency	Sci-Tech Human Resources	Sci-Tech Financial Resources	Sci-Tech Information Resources	Number of Sci-Tech Papers	Number of Patent Applications
Single-threshold test	31.003 ***	39.664 ***	9.026 *	73.094 ***	89.939 **	8.041
	(4.01)	(5.55)	(1.97)	(3.65)	(7.10)	(0.17)
Double-threshold test	56.683 ***	54.337 ***	39.496 ***	46.986 ***	44.382 ***	50.674 ***
	(4.79)	(3.06)	(5.85)	(8.90)	(7.11)	(5.06)
Triple-threshold test	0.000 **	6.714 ***	0.000 *	0.000 *	0.000 *	0.000 *
	(2.23)	(4.45)	(1.96)	(1.83)	(1.69)	(1.78)

Note: The values in parentheses are t-statistics. *, **, *** are significant at the level of 10%, 5% and 1%, respectively.

**Table 3:** Double-threshold estimates.

Model	Threshold Variable	Threshold Estimate 1	95% Confidence Interval	Threshold Estimate 2	95% Confidence Interval
Model (1)	Innovation efficiency	4.952	(4.747, 7.124)	6.966	(6.809, 7.124)
Model (2)	Sci-tech human resources	4.662	(4.766, 5.641)	5.854	(5.641, 5.889)
Model (3)	Sci-tech financial resources	4.530	(4.284, 6.145)	5.069	(4.952, 5.427)
Model (4)	Sci-tech information resources	4.676	(4.284, 5.868)	4.952	(4.676, 4.952)
Model (5)	Number of sci-tech Papers	5.641	(4.284, 5.641)	7.602	(7.602, 7.684)
Model (6)	Number of patent applications	4.676	(4.676, 4.905)	7.757	(7.573, 7.940)

**Table 4:** Double-threshold model parameter estimation results.

Variables	Model (1)	Model (2)	Model (3)	Model (4)	Model (5)	Model (6)
	Innovation Efficiency	Sci-Tech Human Resources	Sci-Tech Financial Resources	Sci-Tech Information Resources	Number of Sci-Tech Papers	Number of Patent Applications
*lnEF_t_*_−1_	1.0237 **	0.9063 ***	0.5247 ***	1.6205 ***	3.2151 ***	0.2754 ***
	(4.09)	(3.35)	(5.68)	(2.99)	(3.97)	(5.18)
*lnEF_it_*_−2_	3.2256 ***	1.7673 ***	0.8699 ***	1.4321 ***	0.2892 ***	1.0275 ***
	(3.27)	(8.22)	(5.75)	(4.89)	(3.60)	(5.13)
X(*T_it_* < *δ*_1_)	−0.1204 ***	0.0405 ***	0.0324 ***	0.0689 ***	0.4441 ***	−0.0443 ***
	(−8.39)	(−4.34)	(6.07)	(9.67)	(−3.54)	(−6.12)
X(*δ*_1_ < *T_it_* < *δ*_2_)	−0.0953 ***	0.0304 ***	0.0595 ***	0.0347 ***	0.0902 ***	−0.0507 ***
	(−8.24)	(3.28)	(4.91)	(9.96)	(−3.65)	(−5.67)
X(*T_it_ > δ*_2_)	−0.0703 ***	0.0470 ***	0.1203 *	0.0450 ***	0.0294 ***	−0.9126 ***
	(−4.38)	(3.87)	(1.67)	(5.00)	(−5.27)	(−3.78)
*lnfdi*	0.0032 ***	0.0928 ***	0.0467 ***	0.0202 **	−0.115 ***	−0.123 ***
	(3.94)	(7.79)	(5.82)	(2.21)	(−8.52)	(−10.16)
*lnthird*	−0.132 ***	−0.00382 ***	−0.0966	−0.0612 **	−0.122 ***	−0.0549 ***
	(−9.55)	(−4.14)	(−1.60)	(−2.08)	(−9.15)	(−5.66)
*lnurbanc*	−0.0033 ***	0.0577 ***	0.0179 **	0.0951 ***	−0.0929 ***	−0.0880 ***
	(−8.45)	(8.77)	(2.27)	(4.29)	(−3.65)	(−5.01)
*lnruralc*	0.6003	0.0195 ***	0.1106 ***	−0.5080	0.0150 **	0.0604
	(1.55)	(4.65)	(11.00)	(−0.83)	(2.24)	(0.55)
*lnedu*	−0.3625	−0.1584	−0.0957	−1.4871	−0.5226	−0.4877
	(1.07)	(0.98)	(1.42)	(0.99)	(1.38)	(1.00)
*lnpollu*	−0.0187 ***	−0.00641	−0.0105	−0.0155 *	−0.0154 ***	−0.0116 **
	(−5.82)	(−0.65)	(−1.42)	(−1.71)	(−6.84)	(−2.18)
C	−0.6080 ***	0.0305 ***	0.3093 ***	0.2080 **	−0.5052 ***	−0.0608 ***
	(−4.66)	(9.40)	(2.79)	(2.10)	(−3.23)	(−4.24)

Note: The values in parentheses are *t* values, *, **, *** are significant at the level of 10%, 5% and 1%.

**Table 5:** Double-threshold estimates of innovative cities.

Model	Threshold Variable	Threshold Estimate 1	95% Confidence Interval	Threshold Estimate 2	95% Confidence Interval
Model (1)	Innovation efficiency	3.025	(2.771, 3.898)	4.767	(3.994, 5.432)
Model (2)	Sci-tech human resources	4.028	(3.726, 4.627)	5.119	(4.728, 5.209)
Model (3)	Sci-tech financial resources	5.066	(4.729, 6.083)	5.970	(5.520, 6.172)
Model (4)	Sci-tech information resources	4.859	(4.265, 5.007)	5.703	(5.580, 6.219)
Model (5)	Number of sci-tech papers	4.229	(3.904, 5.001)	5.261	(4.889, 5.731)
Model (6)	Number of patent applications	5.088	(4.775, 5.645)	7.337	(6.367, 7.558)

**Table 6:** Threshold model parameter estimation results of innovative cities.

Variables	Model (1)	Model (2)	Model (3)	Model (4)	Model (5)	Model (6)
	Innovation Efficiency	Sci-Tech Human Resources	Sci-Tech Financial Resources	Sci-Tech Information Resources	Number of Sci-Tech Papers	Number of Patent Applications
*lnef_it_*_−1_	2.2605 ***	1.7870 ***	0.9430 ***	1.7902 ***	2.5271 ***	0.8540 ***
	(4.28)	(4.09)	(5.15)	(3.78)	(3.65)	(6.44)
*lnef_it_*_−2_	1.8751 ***	1.0695 ***	0.8709 ***	1.0769 ***	0.8740 ***	1.5803 ***
	(3.92)	(4.85)	(7.10)	(4.38)	(3.97)	(4.56)
X(*T_it_* < *δ*_1_)	−0.1749 ***	0.0258 ***	0.0448 ***	0.00240 ***	−0.0623 ***	−0.0361 ***
	(−8.45)	(2.79)	(5.67)	(3.41)	(−5.19)	(−5.11)
X(*δ*_1_ < *T_it_* < *δ*_2_)	−0.2449 ***	0.0328 ***	0.0196 **	0.00952 **	−0.0713 ***	−0.0343 ***
	(−9.60)	(3.83)	(2.51)	(2.23)	(−5.19)	(−4.88)
X(*T_it_ > δ*_2_)	−0.3066 **	0.0315 ***	0.0803 ***	0.106 ***	−0.1100 ***	−0.00604
	(−2.62)	(3.94)	(7.17)	(11.66)	(−9.49)	(−0.55)
*lnfdi*	−0.1051 ***	−0.6021 ***	−0.3004 ***	−0.4508 ***	−0.3025 ***	−0.6016 ***
	(−6.97)	(−5.41)	(−6.07)	(−5.00)	(−3.55)	(−4.84)
*lnthird*	−0.7300 ***	−0.0009 **	−0.00000508	0.002397 ***	−0.0146 *	−0.0036551 ***
	(−7.04)	(−2.20)	(−0.01)	(3.41)	(−1.89)	(−3.78)
*lnurbanc*	0.0062 ***	0.0047 ***	0.080311 ***	0.124489 ***	−0.10963 ***	−0.0060372 ***
	(5.91)	(3.13)	(−4.39)	(10.35)	(−9.53)	(−4.56)
*lnruralc*	0.5511	0.0007	0.00019 ***	0.001948 ***	0.00149 **	0.1041843 ***
	(1.17)	(0.65)	(3.92)	(3.67)	(2.24)	(7.75)
*lnedu*	−0.7958	−0.5541	−0.6835	−0.8814	−1.2070	−0.9587
	(0.57)	(1.01)	(0.65)	(1.07)	(1.44)	(0.88)
*lnpollu*	0.6121 ***	0.0025 ***	−5.08 × 10^−6^	−0.175945 ***	−0.14387 ***	−0.00880068 ***
	(4.63)	(−5.69)	(−0.993)	(−11.85)	(−9.53)	(−5.01)
C	−0.9961 ***	0.9404 ***	0.7957 ***	0.5140 ***	−0.16285 ***	−0.2884 ***
	(−6.86)	(6.70)	(5.11)	(3.20)	(−5.66)	(−6.81)

Note: The values in parentheses are *t* values, *, **, *** are significant at the level of 10%, 5% and 1% respectively.

**Table 7:** Double-threshold Estimates of non-innovative cities.

Model	Threshold Variable	Threshold Estimate 1	95% Confidence Interval	Threshold Estimate 2	95% Confidence Interval
Model (1)	Innovation efficiency	6.038	(4.747, 7.124)	6.945	(6.809, 7.124)
Model (2)	Sci-tech human resources	5.906	(4.766, 5.641)	6.278	(5.641, 5.889)
Model (3)	Sci-tech financial resources	6.028	(5.874, 6.419)	6.569	(4.880, 6.719)
Model (4)	Sci-tech information resources	6.676	(6.218, 7.065)	6.952	(6.676, 7.021)
Model (5)	Number of sci-tech Papers	6.641	(4.284, 5.641)	7.202	(6.886, 7.690)
Model (6)	Number of patent applications	6.676	(6.506, 6.923)	7.757	(7.501, 7.978)

**Table 8:** Double-threshold model parameter estimation results of non-innovative cities.

Variables	Model (1)	Model (2)	Model (3)	Model (4)	Model (5)	Model (6)
	Innovation Efficiency	Sci-Tech Human Resources	Sci-Tech Financial Resources	Sci-Tech Information Resources	Number of Sci-Tech Papers	Number of Patent Applications
*lnef_it_*_−1_	0.0865 ***	0.7623 **	0.6255 ***	1.0275 ***	2.0501 ***	0.6703 ***
	(5.68)	(2.35)	(4.81)	(3.05)	(3.70)	(4.29)
*lnef_it_*_−2_	2.4786 ***	1.2480 ***	0.9239 ***	1.0170 ***	0.5832 ***	1.5980 ***
	(3.20)	(6.29)	(5.11)	(3.85)	(4.75)	(3.94)
X(*T_it_* < *δ*_1_)	–0.3002 ***	0.0098 ***	0.0045 ***	0.0070 ***	0.0845 ***	–0.1005 ***
	(–6.62)	(3.18)	(4.27)	(3.00)	(–5.49)	(–3.37)
X(*δ*_1_ < *T_it_* < *δ*_2_)	–0.1569 ***	0.0705 ***	0.7268 **	0.5602 **	0.6790 ***	–0.0906 ***
	(–4.88)	(3.56)	(1.99)	(2.48)	(–4.23)	(–3.08)
X(*T_it_ > δ*_2_)	–0.0980 ***	0.0974 ***	0.8593 ***	0.6096 ***	0.7180 ***	–0.0704 **
	(–6.05)	(3.75)	(6.49)	(8.17)	(–5.02)	(–2.26)
*lnfdi*	0.0007 ***	0.0422 ***	0.0064 ***	0.1048 ***	0.1562 ***	0.6019 ***
	(3.55)	(4.90)	(6.50)	(5.83)	(4.06)	(3.92)
*lnthird*	–0.0098 ***	–0.0147 **	–0.0158	0.0027 ***	–0.0109 *	–0.3051 ***
	(–6.25)	(–2.38)	(–0.49)	(3.77)	(–1.69)	(–3.80)
*lnurbanc*	1.0092 ***	0.9368 ***	0.8030 ***	0.4126 ***	–0.1960 ***	–0.6552 ***
	(4.76)	(3.92)	(–4.58)	(9.03)	(–6.77)	(–4.12)
*lnruralc*	0.7039	0.0657	0.0024 ***	0.0724 ***	0.4027 **	0.8413 ***
	(1.09)	(0.88)	(3.39)	(4.18)	(2.13)	(6.56)
*lnedu*	–0.0021	–0.0436	–0.0289	–0.4671	–0.5062	–0.0945
	(1.01)	(0.75)	(0.94)	(1.23)	(1.70)	(1.09)
*lnpollu*	–0.0167 ***	–0.0943 ***	–0.1290	–0.4755 ***	–0.8137 ***	–0.4068 ***
	(5.08)	(–5.37)	(–0.65)	(–7.49)	(–6.26)	(–4.87)
C	–4.5780 ***	3.6903 ***	0.8896 ***	1.5630 ***	–0.8905 ***	–0.6759 ***
	(–3.79)	(5.08)	(4.49)	(3.80)	(–5.29)	(–4.24)

Note: The values in parentheses are *t* values, *, **, *** are significant at the level of 10%, 5% and 1% respectively.

## Data Availability

Due to the confidentiality and privacy of the data, they will only be provided upon reasonable request.

## References

[1] Rees W.E. (1992). Ecological footprint and appropriated carrying capacity: What urban economics leaves out. Environ. Urban.

[2] Wackernagel M., Onisto L., Bello P., Linares A.C. (1999). National natural capital accounting with the ecological footprint concept. Ecol. Econ..

[3] Safitri S., Wikantika K., Riqqi A., Deliar A., Sumarto I. (2021). Spatial Allocation Based onPhysiological Needs and LandSuitability Using the Combination ofEcological Footprint and SVM (CaseStudy: Java Island, Indonesia). ISPRS Int. J. Geo-Inf..

[4] Prince N.S. (2021). Natural resources, urbanisation, economic growth and the ecological footprint in South Africa: The moderating role of human capital. Quaest. Geogr..

[5] Dhiaf M.M., Najaf K., Marashdeh H., Atayah O.F., Frederico G.F. (2021). The Role of Project’s Initiatives Focused on the Reduction of Environmental Footprints during COVID-19: Evidence from the United States Firms. Oper. Manag. Res..

[6] Zailani S., Amran A., Jumadi H. (2011). Green innovation adoption among logistics service providers in malaysia: An exploratory study on the managers perceptions. Int. Bus. Manag..

[7] Ongsakul V., Chatjuthamard P., Jiraporn P. (2022). Does the market for corporate control impede or promote corporate innovation efficiency? Evidence from research quotient. Financ. Res. Lett..

[8] Fan F., Zhang K.K., Dai S.Z. (2021). Decoupling analysis and rebound effect between China’s urban innovation capability and resource consumption. Technol. Anal. Strateg. Manag..

[9] Ke H., Dai S., Fan F. (2021). Does innovation efficiency inhibit the ecological footprint? An empirical study of China’s provincial regions. Technol. Anal. Strateg. Manag..

[10] Li Z., Liu F.S. (2021). Has the Pilot Project of Innovative Cities Increaseed the Level of Innovation?. Soc. Sci. Res..

[11] Zhang Y. (2021). Has the Pilot Project of Innovative Cities Increased the Level of Scientific and Technological Talents Agglomeration: A Quasi Experimental Study Based on 240 Cities in China. Sci. Technol. Prog. Policy.

[12] Kaklauskas A., Herrera-Viedma E., Ec Henique V., Zavadskas E.K., Podviezko A. (2020). Multiple criteria analysis of environmental sustainability and quality of life in post-soviet states. Ecol. Indic..

[13] Global Footprint Network Footprint for Nations (Data Downloads, Licenses, and Support). (EB/OL). GFN. https://www.footprintnetwork.org/licenses/.

[14] Jia J., Deng H., Jing D., Zhao J. (2009). Analysis of the major drivers of the ecological footprint using the stirpat model and the pls method--a case study in henan province, china. Ecol. Econ..

[15] Chen B., Chen G.Q. (2007). Modified ecological footprint accounting and analysis based on embodied exergy-a case study of the chinese society 1981–2001. Ecol. Econ..

[16] Kabir M., Habiba U.E., Shafiq M. (2021). Green revolution: An innovation for environmental pollution in changing climate of world. Sci. Proc. Ser..

[17] Yang Q., Gao D., Song D., Li Y. (2021). Environmental regulation, pollution reduction and green innovation: The case of the Chinese water ecological civilization city pilot policy. Econ. Syst..

[18] Grossman G., Krueger A. (1995). Economic growth and the environment. Q. J. Econ..

[19] Porter M.E. (1990). Competitive Advantage of Nations? The Competitive Advantage of Nations.

[20] Fang C., Ma H., Wang Z., Li G. (2014). Comprehensive assessment and spatial heterogeneity of the construction of innovative cities in China. Acta Geogr. Sin..

[21] Zhang S., Wang X., Zhang B. (2021). The policy effects of innovative city pilot on the dual efficiency of industry-university-research knowledge flow. Technol. Anal. Strateg. Manag..

[22] Malthus T., Johnson J. (1798). An Essay on the Principle of Population.

[23] Commoner B. (1971). The Closing Circle: Confronting the Environmental Crisis.

[24] Ehrlich P.R., Holdren J.P. (1971). Impact of population growth. Science.

[25] Scheurer J. (2001). Urban Ecology, Innovations in Housing Policy and the Future of Cities: Towards Sustainability in Neighbourhood Communities. Ph.D. Thesis.

[26] Ahern J., Cilliers S., Niemela J. (2014). The concept of ecosystem services in adaptive urban planning and design: A framework for supporting innovation. Landsc. Urban Plan..

[27] World Wide Fund for Nature (2015). China’s Ecological Footprint Report.

[28] Han J.Y., Ha S.T., Cho S.P. (2020). The impact of innovative efficiency on performance of firms. Korea Soc. Innov. Manag. Econ..

[29] Fan F., Zhang X.R. (2021). Transformation effect of resource-based cities based on PSM-DID model: An empirical analysis from China. Environ. Impact Assess. Rev..

[30] Fan F., Dai S.Z., Zhang K.K. (2021). Innovation agglomeration and urban hierarchy: Evidence from Chinese cities. Appl. Econ..

[31] Yu H., Zhang J., Zhang M., Fan F. (2021). Cross-national knowledge transfer, absorptive capacity, and total factor productivity: The intermediary effect test of international technology spillover. Technol. Anal. Strateg. Manag..

[32] Hu Y., Cao X., Chen J. (2021). Correcting the saturation effect in dmsp/ols stable nighttime light products based on radiance-calibrated data. IEEE Trans. Geosci. Remote Sens..

[33] Zhang X., Gibson J. (2022). Using Multi-Source Nighttime Lights Data to Proxy for County-Level Economic Activity in China from 2012 to 2019. Remote Sens..

[34] Shi Y., Shao C., Zhang Z. (2020). Efficiency and driving factors of green development of tourist cities based on ecological footprint. Sustainability.

[35] York R., Rosa E.A., Dietz T. (2003). STIRPAT, IPAT and ImPACT: Analytic tools for unpacking the driving forces of environmental impacts. Ecol. Econ..

[36] Ahmed Z., Zhang B., Cary M. (2020). Linking economic globalization, economic growth, financial development, and ecological footprint: Evidence from symmetric and asymmetric ardl. Ecol. Indic..

[37] Rehman A., Radulescu M., Ma H., Dagar V., Hussain I., Khan M.K. (2021). The impact of globalization, energy use, and trade on ecological footprint in pakistan: Does environmental sustainability exist?. Energies.

[38] Ke H., Dai S., Yu H. (2021). Spatial effect of innovation efficiency on ecological footprint: City-level empirical evidence from china. Environ. Technol. Innov..

[39] China National Knowledge Infrastructure (EB/OL). CNKI. https://data.cnki.net/Yearbook/Navi?type=type&code=A.

[40] Hansen B.E. (1999). Threshold effects in non-dynamic panels: Estimation, testing, and inference. J. Econom..

[41] Kremer D., Münster M., Freitag M., Stadler S., Schletz A. Emerging Service Organization in Manufacturing Enterprises: A Dynamic Pathway Model of Servitization. Proceedings of the XXIII International RESER Conference.

[42] Kremer S., Bick A., Nautz D. (2009). Inflation and growth: New evidence from a dynamic panel threshold analysis. SFB 649 Discuss. Pap..

[43] Dietz T., Rosa E.A. (1994). Rethinking the environmental impacts of population, affluence and technology. Hum. Ecol. Rev..

[44] Rizwana Y., Cui Z.H., Wasi H.S., Muhammad A.K., Anwar K. (2022). Exploring the role of biomass energy consumption, ecological footprint through FDI and technological innovation in B&R economies: A simultaneous equation approach. Energy.

[45] Yang B., Jahanger A., Ali M. (2022). Remittance inflows affect the ecological footprint in BICS countries: Do technological innovation and financial development matter?. Environ. Sci. Pollut. Res..

[46] Ahmad M., Jiang P., Majeed A., Umar M., Khan Z., Muhammad S. (2020). The dynamic impact of natural resources 2021, technological innovations and economic growth on ecological footprint: An advanced panel data estimation. Resour. Policy.

[47] Usman M., Hammar N. (2021). Dynamic relationship between technological innovations, financial development, renewable energy, and ecological footprint: Fresh insights based on the STIRPAT model for Asia Pacific Economic Cooperation countries. Environ. Sci. Pollut. Res..

[48] Destek M.A., Manga M. (2021). Technological innovation, financialization, and ecological footprint: Evidence from BEM economies. Environ. Sci. Pollut. Res..

